# What procedures must any cath lab in Africa offer?

**Published:** 2016

## Abstract

Answering this question, Dr Francois Bourlon from Monaco named these as some of the key procedures any African cath lab should offer:

right- and left-sided pressure studies
coronary angiography
percutaneous coronary intervention (notably with radial access)
mitral/pulmonary valvuloplasty
PDA closure
atrial septostomy
pacing, both permanent and temporary
pericardiocentesis
bilateral iliac intervention.

This is an ideal scenario and Africa as a whole is still a long way from achieving it. As Dr Bourlon pointed out, facilities vary greatly across the continent. ‘There are a few cath labs that are well equipped and manned by well-trained staff. Many others have extremely limited resources, however, requiring interventional cardiologists to be versatile and skilful. And in many parts of Africa, a cath lab is just a dream.’

## A Tanzanian success story

But dreams can be achieved. Dr Robert Mvungi, from Tanzania, shared an inspiring update on what his cath lab has accomplished in the past year since AfricaPCR 2015. Established in Dar-es-Salaam in 2013, it is the country’s first cath lab. Its first procedure, a permanent pacemaker implantation, took place on 19 November 2013 and the first coronary angiogram was performed on 25 June 2014.

Dr Mvungi noted that there were challenges in equipping the facility and training the team. The cath lab nurses had to be trained in India, and returned with good skills. To date the laboratory has undertaken 365 adult procedures and 85 paediatric ones, most of the latter being PDA closures. Overall, many more procedures were undertaken in 2015 than in 2013/2014 – these included pacemaker procedures, stenting, right heart studies and fluoroscopies. ‘We’ve also done eight percutaneous mitral balloon valvuloplasties to date’, he told delegates at AfricaPCR 2016. Coronary artery bypass grafting procedures are now performed onsite.

There are many plans in view to ensure the laboratory goes from strength to strength. ‘We’re establishing a database registry to monitor ongoing quality improvement and focusing on building human resource capacity. I’m also looking forward to our acquiring new equipment that will enable us to undertake more complex procedures in the future.’

He believes the following are critical elements for establishing a cath lab in Africa:

government support (73% of the laboratory’s work is funded by Tanzania’s national health insurance)
human resource capacity
adequate funding
a continuous supply of consumables
a fluoroscopy and haemodynamic system
supporting equipment
maintenance services.

He concluded by underscoring the importance of institutional relationships. These will allow African cardiologists to train with recognised leaders internationally and then return to their own countries with the skills necessary to run an autonomous African cath lab.

